# Continuous attractor network models of grid cell firing based on excitatory–inhibitory interactions

**DOI:** 10.1113/JP270630

**Published:** 2016-02-24

**Authors:** Oliver Shipston‐Sharman, Lukas Solanka, Matthew F. Nolan

**Affiliations:** ^1^ Centre for Integrative PhysiologyUniversity of EdinburghHugh Robson BuildingEdinburghEH8 9XDUK

## Abstract

Neurons in the medial entorhinal cortex encode location through spatial firing fields that have a grid‐like organisation. The challenge of identifying mechanisms for grid firing has been addressed through experimental and theoretical investigations of medial entorhinal circuits. Here, we discuss evidence for continuous attractor network models that account for grid firing by synaptic interactions between excitatory and inhibitory cells. These models assume that grid‐like firing patterns are the result of computation of location from velocity inputs, with additional spatial input required to oppose drift in the attractor state. We focus on properties of continuous attractor networks that are revealed by explicitly considering excitatory and inhibitory neurons, their connectivity and their membrane potential dynamics. Models at this level of detail can account for theta‐nested gamma oscillations as well as grid firing, predict spatial firing of interneurons as well as excitatory cells, show how gamma oscillations can be modulated independently from spatial computations, reveal critical roles for neuronal noise, and demonstrate that only a subset of excitatory cells in a network need have grid‐like firing fields. Evaluating experimental data against predictions from detailed network models will be important for establishing the mechanisms mediating grid firing.

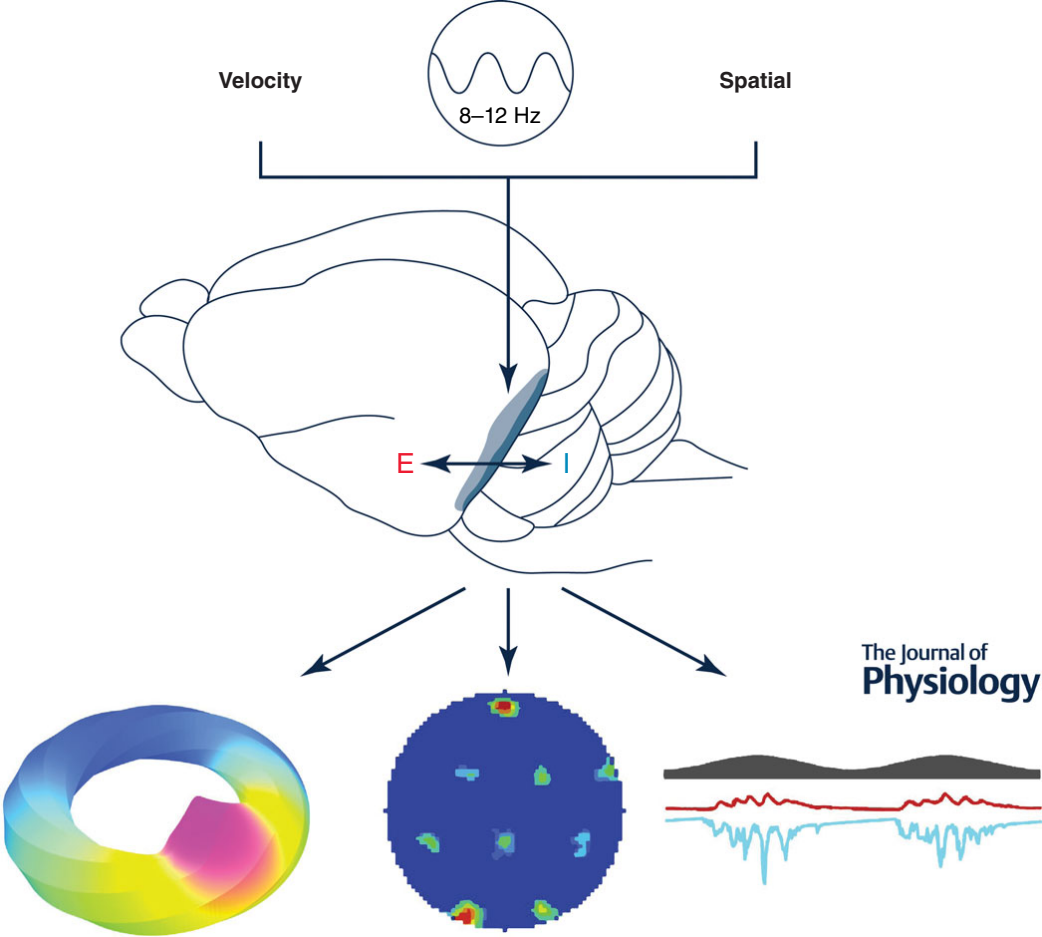

**Abbreviations**
E–I: excitatory–inhibitory
L2PCs: layer 2 pyramidal cells
L2SCs: layer 2 stellate cells
MEC: medial entorhinal cortex

## Introduction

Neural representations of space within the hippocampus and medial entorhinal cortex (MEC) are critical for navigation and memory. Grid cells in the MEC have firing fields that encode position using an allocentric, regular triangular matrix or *grid*‐like firing pattern (Hafting *et al*. 2005). Grid representations have the properties of a high capacity, high resolution and error correcting code for self‐localisation (Fiete *et al*. 2008; Sreenivasan & Fiete, 2011; Mathis *et al*. 2012). The spatially periodic features of grid firing fields have led to the view that they are the output of computation by a path integrator that translates self‐motion signals into estimates of location (McNaughton *et al*. 2006). In this review, we will consider evidence that network attractor dynamics arising from excitatory–inhibitory interactions account for grid firing patterns within MEC circuits.

The organisation within the MEC of spatial firing properties is an important constraint on mechanistic models for grid firing. Grid cells form networks in anatomically overlapping but functionally discrete modules, with cells of the same module sharing their grid spacing and orientation but having randomly distributed phases (relative offset of grid apices) (Hafting *et al*. 2005; Barry *et al*. 2007; Stensola *et al*. 2012). The highest density of grid cells is in layer 2 of the MEC (Sargolini *et al*. 2006). Grid cells in this layer also show the greatest prospective bias in their code for location (Kropff *et al*. 2015). There are two major populations of excitatory cells in this layer. Neurons positive for the marker reelin have stellate morphology and project to the dentate gyrus of the hippocampus (Klink & Alonso, 1997; Varga *et al*. 2010), while neurons positive for calbindin have a more pyramidal morphology and project to the CA1 region of the hippocampus (Varga *et al*. 2010; Kitamura *et al*. 2014; Ray *et al*. 2014). We will refer to these cell populations as layer 2 stellate cells (L2SCs) and layer 2 pyramidal cells (L2PCs), respectively (Klink & Alonso, 1997) (L2SCs and L2PCs have also been referred to as ‘Ocean’ and ‘Island’ cells; Kitamura *et al*. 2014). Both L2SCs and L2PCs may have grid firing fields, although the majority of neurons in each population do not appear to generate typical grid firing patterns (Tang *et al*. 2014; Sun *et al*. 2015). During behaviours that produce grid firing, neurons in superficial layers of the MEC also generate fast gamma frequency (60–140 Hz) oscillations that are modulated by the slower theta rhythm (Chrobak & Buzsaki, 1998; Colgin *et al*. 2009). While all grid cells encode location through their firing rate, some also represent location through timing of their action potentials relative to the network theta rhythm (Hafting *et al*. 2008; Reifenstein *et al*. 2012).

**Figure 1:**
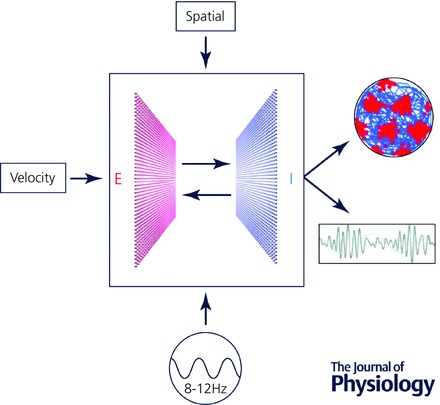
**Components of a generic E–I model for generation of grid firing and nested gamma oscillations** Integration of velocity input by continuous attractor networks built from interacting excitatory and inhibitory neurons can generate grid firing fields. When the networks receive a theta modulated input they generate gamma frequency output that is modulated at theta frequency. A spatial input is required to oppose drift in the grid representation. Data are from Pastoll *et al*. (2013).

Several conceptual models have been proposed to explain grid firing patterns (for reviews see Burgess & O'Keefe, 2011; Giocomo *et al*. 2011; Zilli, 2012). However, implementing models in ways that are consistent with the biophysics and connectivity of entorhinal neurons is challenging (Remme *et al*. 2010; Pastoll *et al*. 2012). Here, we will explore insights from models in which grid‐like firing patterns emerge as a result of path integration in continuous attractor networks composed of excitatory and inhibitory neurons, with membrane potential dynamics that approximate real neurons (Fig. 1). We will argue that this class of models is particularly useful as they can be constrained by experimentally measured synaptic connectivity and oscillatory network activity, as well as by action potential firing during spatial behaviours. They therefore generate specific predictions that are testable by diverse experimental approaches from anatomical analysis through to electrophysiological recordings of single cell and network activity.

## Continuous attractor networks as models for grid generation

**Figure 2:**
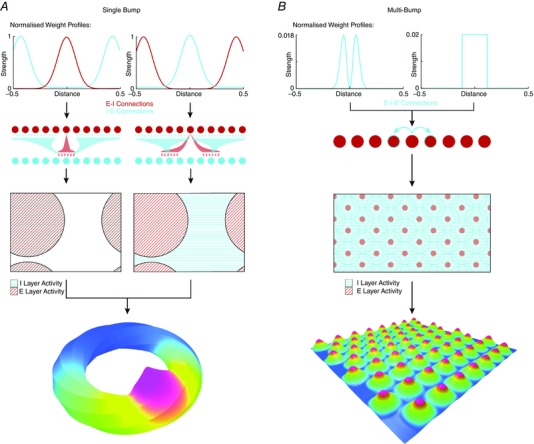
**Single‐ and multi‐bump attractor models of grid firing have distinct circuit organisation** *A*, In single bump models grid firing of excitatory cells can be generated by synaptic profiles that produce either surround excitation or surround inhibition. The surround connectivity is strongest for connections to neurons at a distance of about one‐half the width of the sheet. Each neuron makes divergent connections to many target neurons, and receives convergent input from many presynaptic neurons. *B*, In multi‐bump networks the strongest connections are onto neurons at a much shorter distance relative to the size of the sheet. The upper graphs plot synaptic strength as a function of position in the neural sheet, which is given a width of one. The plots below schematise the resulting E–I connectivity, illustrate the organisation of activity in the neural sheet and the organisation of excitatory cell activity in three dimensions. The connectivity profiles shown for the multi‐bump models are based on networks containing only inhibitory neurons, with either surround inhibition (Burak & Fiete, 2009) or local inhibition (Couey *et al*. 2013). The networks could be considered as having dedicated interneurons receiving input from each excitatory neuron.

Continuous attractor networks are dynamical systems whose intrinsic properties drive activity towards a stable state; this can be visualised in a state space comprising an energy surface upon which stable states are represented by low energy regions (Brody *et al*. 2003). States existing outwith these regions will decay ‘downwards’ towards the low energy points. A network's intrinsic connections can be configured so its preferred states will correspond to localised bumps of activity. Mathematical functions can then be implemented in the network's state space by movement of the bumps of activity in response to inputs to the network (Conklin & Eliasmith, 2005; Eliasmith, 2005). In continuous attractor network models of spatial coding, the computation performed is integration of velocity input to generate an estimate of location relative to a known start point, referred to as path integration (McNaughton *et al*. 1996, 2006; Zhang, 1996; Samsonovich & McNaughton, 1997). Such networks do not necessarily generate triangular grid‐like firing fields, but can do so with appropriately configured connections. In networks that model grid firing, stable states manifest either as a bump (Fig. 2 *A*) or as multiple bumps of activity (Fig. 2 *B*) on a two‐dimensional sheet of phase‐arranged grid cells (Fuhs & Touretzky, 2006; Guanella *et al*. 2007). Given velocity inputs the activity bump(s) represent movement in space by propagating across the sheet. This mechanism for path integration can be implemented by networks in which individual grid cells receive velocity inputs tuned to a particular movement direction, with the local connections of each grid cell offset so that an increase in its input will tend to push the activity bump in an appropriate direction across the neural sheet (Fuhs & Touretzky, 2006; Guanella *et al*. 2007; Burak & Fiete, 2009). Alternatively, path integration could be achieved through interactions between a layer of heading‐independent grid cells and multiple layers of head direction‐modulated grid cells, which each integrate a single head direction input with speed signals and feedback from the heading‐independent grid layer (Samsonovich & McNaughton, 1997). While the latter class of models require many more neurons to account for path integration, because separate layers are required for each heading direction, they have the advantage that they naturally account for direction modulated (or conjunctive) grid cells as well as pure grid cells.

Continuous attractor network models have been implemented at various levels of detail and a close correspondence to known neural connectivity or dynamics is not necessary to generate grid‐like firing fields. Indeed, experimental observations have corroborated a number of generic predictions that are independent of the details of the circuitry used for model implementation (McNaughton *et al*. 2006). (1) Populations of grid cells are organised into modules in which each neuron has a common spatial phase and orientation (Stensola *et al*. 2012). (2) The spatial phase relationship between cells is maintained even following environmental manipulations that restructure the spatial firing pattern of individual cells (Yoon *et al*. 2013). (3) The envelope of the membrane potential of grid cells changes slowly on entry to and exit from their firing fields (Domnisoru *et al*. 2013; Schmidt‐Hieber & Hausser, 2013). (4) Removal of excitatory drive causes cells that previously had grid fields to encode head direction (Bonnevie *et al*. 2013), which is consistent with movement of activity bumps in continuous attractor networks relying on each grid cell receiving a tuned head direction input (Fuhs & Touretzky, 2006; Guanella *et al*. 2007; Burak & Fiete, 2009; Bonnevie *et al*. 2013; Pastoll *et al*. 2013).

While these observations are consistent with continuous attractor network models accounting for rate coded grid fields, most existing models do not readily account for precession in the timing of action potentials fired by some grid cells relative to the theta rhythm (Hafting *et al*. 2008; Reifenstein *et al*. 2012). One‐dimensional attractor networks, based on interaction between a direction‐independent cell population and direction modulated cell populations, can generate repeating firing fields and phase precession (Navratilova *et al*. 2012). Extension of this mechanism to two dimensions will require additional neuronal layers for each heading direction (Samsonovich & McNaughton, 1997). An alternative is that phase precession and attractor states are established independently. For example, phase precession can be explained by hybrid models that include a mechanism for grid firing based on interference between oscillations (Burgess *et al*. 2007; Hasselmo *et al*. 2007), in addition to mechanisms for generation of network attractor states (Schmidt‐Hieber & Hausser, 2013; Bush & Burgess, 2014).

## Emergence of attractor states through excitatory–inhibitory interactions

How might attractor mechanisms for grid firing be implemented in networks of neurons? Do the properties of neural circuitry in the MEC constrain models or lead to predictions that distinguish between different models? Grid computation in continuous attractor networks requires emergence of stable bumps of activity. This can be achieved using reduced models in which separate populations of excitatory and inhibitory neurons are not explicitly considered. In these models, either each neuron locally excites nearby neurons and inhibits more distant neurons (Fuhs & Touretzky, 2006), or spatially structured inhibitory connections act in concert with excitatory drive to the whole network (Burak & Fiete, 2009; Couey *et al*. 2013). However, use of local excitatory connections is inconsistent with evidence that L2SCs are not directly connected to one another (Dhillon & Jones, 2000; Couey *et al*. 2013; Pastoll *et al*. 2013), but instead interact indirectly via inhibitory interneurons (Couey *et al*. 2013; Pastoll *et al*. 2013). Moreover, because grid cells are excitatory neurons, an inhibitory output from grid cells is inevitably an over‐simplification. One could address this by assuming that the inhibitory output from grid cells is equivalent to an excitatory connection to a dedicated inhibitory interneuron. However, this is inconsistent with convergent (many to one) and divergent (one to many) connectivity between excitatory and inhibitory networks (Couey *et al*. 2013), and with there being many more excitatory than inhibitory neurons in layer 2 of the MEC (Canto *et al*. 2008). Thus, while offering conceptually important explanations for grid firing, reduced models are limited in their ability to evaluate consequences of experimentally determined connectivity.

Models that explicitly consider interactions between separate populations of excitatory and inhibitory neurons inevitably differ from reduced models, leading to new insights and predictions (Pastoll *et al*. 2013; Widloski & Fiete, 2014; Solanka *et al*. 2015). Given appropriately structured network connectivity these excitatory–inhibitory (E–I) models generate network attractor states (Fig. 2). Structured connectivity can be implemented by varying the strength of connections between neurons according to their position in the network, while maintaining a fixed probability of a connection being present (Pastoll *et al*. 2013; Widloski & Fiete, 2014; Solanka *et al*. 2015). Alternatively, synaptic strength can remain fixed but the probability of connections varied as a function of distance between pre‐ and postsynaptic neurons on the neural sheet (Solanka *et al*. 2015). Evidence that the amplitude of inhibitory inputs to stellate cells has a bimodal distribution is consistent with structuring of connection probability rather than the strength of connections (Couey *et al*. 2013). Models based on E–I interactions also demonstrate that velocity inputs, which are required for movement‐dependent translation of their activity bumps, may target either interneurons or excitatory cells (Pastoll *et al*. 2013). While spatial firing of cells with inhibitory output is implicit in reduced models, in E–I models interneurons have spatial firing fields that depend on the wiring of the network. For example, either surround inhibition or surround excitation supports grid firing by excitatory cells, but in the latter case interneurons have inverted grid fields, whereas in the former they have grid‐like fields (Pastoll *et al*. 2013).

Two important recent experimental studies introduce challenges beyond simply accounting for grid firing by excitatory cells. First, while the firing fields of parvalbumin‐positive interneurons have significant spatial stability, they typically have grid scores below the threshold for grid firing, only rarely appear to have a clear grid‐like organisation (Buetfering *et al*. 2014), and on visual inspection also do not appear to have inverted firing fields, although this is difficult to establish quantitatively. Second, when layer 2 cells are imaged in freely moving animals, only about 10% of identified L2SCs and L2PCs have grid‐like firing fields (Sun *et al*. 2015). This is surprising given that neurons within each population appear to have similar synaptic connectivity and intrinsic properties. This could suggest that the grid firing neurons correspond to sub‐groups of cells with distinct, but not yet identified, cellular or circuit properties. Otherwise, models for grid firing must explain how grid patterns are produced by only a subset of neurons that at a cellular and circuit level are indistinguishable from non‐grid cells.

**Figure 3:**
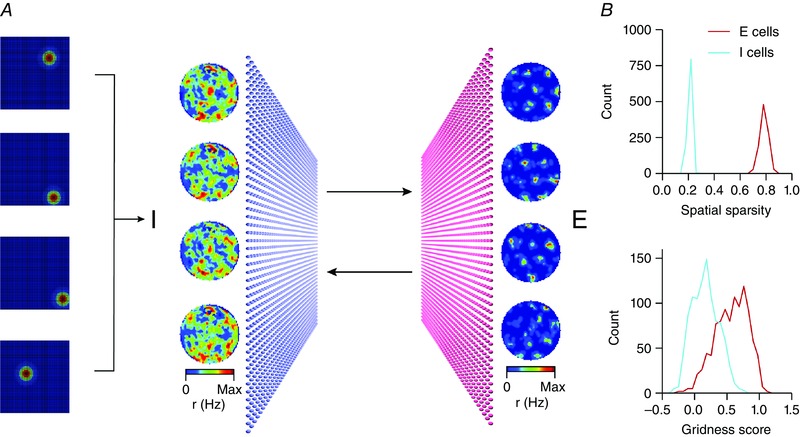
**Spatial firing of interneurons in E–I attractor models** *A*, schematic organisation of an E–I network with additional random place field inputs to each interneuron (left). Example firing fields of I cells (middle) and E cells (right) are shown adjacent to the schematised neurons. *B*, histograms of the spatial sparsity (upper) and gridness score (lower) for E–I networks simulated as in *A*. Note that most interneurons and many excitatory cells have grid scores <0.5. Data are from Solanka *et al*. 2015. r in “r(Hz)” is spike rate.

These challenges may be addressed using E–I networks and by considering that *in vivo* entorhinal neurons may receive spatial signals that can be considered as noise in the sense that they are not used to promote grid firing. Thus, when E–I models are extended to include random spatial input to interneurons, excitatory neurons in these networks continue to generate grid‐like firing fields, but the hexagonal symmetry of interneuron firing fields is reduced (Fig. 3) (Solanka *et al*. 2015). In these networks the fraction of excitatory and inhibitory cells classified as grid cells drops substantially, with almost no interneurons classified as having grid fields (Fig. 3). Thus, the finding that only a subset of layer 2 cells has grid‐like firing fields need not imply that grid and non‐grid cells are distinguished by distinct cellular or circuit properties, while the absence of a clear grid signature in the firing of individual interneurons may nevertheless be compatible with models based on E–I interactions.

## Single‐ and multi‐bump networks differ in their local and long‐range connectivity

Continuous attractor network models for grid firing exist in versions that differ in their number of activity bumps. These functional differences result primarily from connections spanning different distances relative to the size of the network.

In single bump networks (also referred to as periodic networks, cf. Widlowski & Fiete, 2015) the planar attractor manifold is wrapped into a torus (Samsonovich & McNaughton, 1997; Guanella *et al*. 2007; Pastoll *et al*. 2013). This conceptual torus structure is actuated in the synaptic connectivity of the network, with cells on one edge of the sheet connected to those on the opposite side (Fig. 2 *A*). When an animal travels continuously in one direction the activity bump moves periodically around the network. Generation of a triangular rather than rectangular organisation of grid fields is dependent on the addition of a phase shift in one axis resulting in a twisted torus attractor manifold (Fig. 2 *A*).

Networks with multiple bumps of activity also have their neurons arranged on a two‐dimensional manifold; however, a hexagonal population activity bump organisation arises from the most energetically efficient packing of the rings of inhibition; each circle of inhibition repels neighbouring circles to a maximal distance until stabilising into a grid of activity bumps (Fig. 2 *B*) (Fuhs & Touretzky, 2006; Burak & Fiete, 2009; Couey *et al*. 2013). During movement the bumps of activity propagate across the network and individual neurons generate grid firing patterns. Multi‐bump networks can either be implemented with periodic boundaries (also referred to as partially periodic networks), so that, much as in single bump models, the activity bump wraps to the other side of the network, or they can have boundaries (also referred to as aperiodic networks). In this case, when bumps reach the edge of the network they disappear, while on the opposite side of the network local competitive synaptic interactions cause new bumps to spontaneously form as existing bumps move away (Fuhs & Touretzky, 2006; Burak & Fiete, 2009).

When single bump attractors are implemented in E–I networks, each neuron's connections extend over a relatively large fraction of the network (Fig. 2 *A*). Thus, neurons making surround connections have their highest connection probability, or connection strength, with neurons at a distance of approximately half the width of the untwisted neural sheet. This distance refers to separation based on the order of connectivity in the network rather than anatomical distance (cf. Widloski & Fiete, 2014). Indeed the anatomical organisation of cell bodies of neurons with repeating firing fields appears relatively weak compared to the organisation of neural sheets in continuous attractor network models (Heys *et al*. 2014), suggesting that synaptic connectivity required for grid firing can be established without ordering of neuronal cell bodies (Widloski & Fiete, 2014). In contrast to single bump networks, the connectivity in multi‐bump attractors is much more localised relative to the overall size of the network (Fig. 2 *B*). This suggests that quantification of the extent of connectivity between excitatory and inhibitory neurons could be used to distinguish between single‐ and multi‐bump models. Local circuit perturbations through thermo‐ or chemomodulation in conjunction with multi‐unit recordings might also distinguish between single‐ and multi‐bump networks (Widloski & Fiete, 2015).

## Excitatory–inhibitory interactions provide a common mechanism for grid firing and network oscillations

Successful models of brain circuits should account for network dynamics as well as the firing patterns of individual cells. Dynamics can be modelled by simulating networks of integrate and fire neurons. In these models synaptic input to a neuron charges its membrane capacitance, which is in turn discharged through a resistance. Action potentials occur when the membrane potential crosses a threshold. In exponential integrate and fire neurons the spike threshold has been replaced with an exponential function in order to obtain more realistic spike initiation dynamics (Fourcaud‐Trocme *et al*. 2003). Although integrate and fire models neglect details of morphology and ion channel biophysics, their dynamics are a good approximation for physiological synaptic integration, making them an important bridge between abstract theoretical and more detailed cellular models.

**Figure 4:**
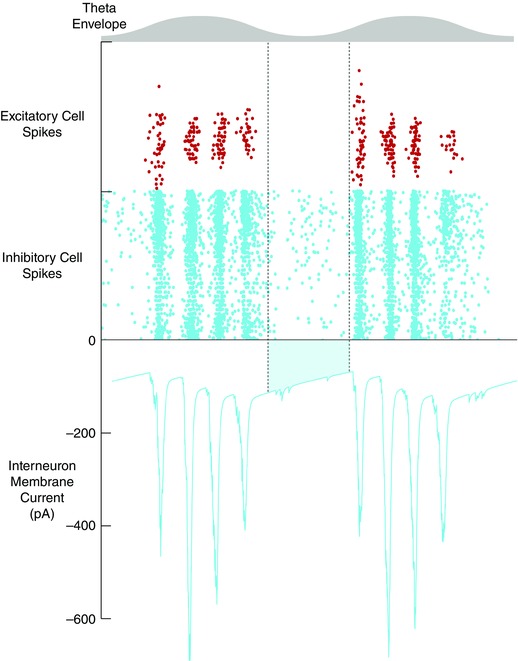
**Theta nested gamma activity in E–I models** Spike rasters for E cells (red) and I cells (blue) during two theta cycles (grey). The excitatory synaptic input to a representative I cell is illustrated below. Note that a substantial residual inward current (blue shading) is maintained during the phase of the theta oscillation when spike activity of excitatory cells is reduced. The residual current enables the bump of activity to be maintained across theta cycles. Data are from Pastoll *et al*. (2013) and Solanka *et al*. (2015).

Models of interacting populations of excitatory and inhibitory exponential integrate and fire neurons can account for both grid firing and gamma oscillations (Pastoll *et al*. 2013; Solanka *et al*. 2015). When the models receive theta modulated input the gamma oscillations are nested at a fixed phase within each theta cycle (Fig. 4). This is consistent with experimental findings that theta modulated optogenetic activation of layer 2 circuits is sufficient to generate nested gamma activity resembling that observed in behaving animals (Chrobak & Buzsaki, 1998; Pastoll *et al*. 2013). In these experiments, and in the corresponding models, gamma oscillations emerge through fast time scale E–I interactions. On each gamma cycle a subset of excitatory neurons fire action potentials. Because the output from each excitatory neuron diverges to many interneurons (Fig. 2 *A*), and as each interneuron receives convergent input from many excitatory cells (Fig. 2 *A*), this output is sufficient to rapidly trigger action potentials in a large fraction of interneurons. Divergent projections from interneurons send inhibitory feedback to excitatory cells, including those that did not spike. A second gamma cycle is initiated on recovery from this inhibition. The divergent connectivity effectively implements a competitive mechanism that limits the number of excitatory cells active on each theta cycle (Tiesinga & Sejnowski, 2009). While E–I models account for both rate coded firing and nested gamma oscillations, a possible limitation of existing models is that theta input is implemented as a common drive to E and I cells. In contrast, only interneurons in the MEC appear to receive inhibitory pacemaker input from the medial septum (Gonzalez‐Sulser *et al*. 2014) while the origin of excitation during theta is currently unknown.

Models that account for grid firing and nested gamma oscillations exclusively through E–I interactions have been extended to incorporate additional features of MEC circuitry (Solanka *et al*. 2015). Experimental observations indicate that inhibitory neurons in layer 2 of the MEC may synapse with one another (Pastoll *et al*. 2013). Addition to E–I models of connections between interneurons stabilises grid firing and increases the frequency of nested gamma oscillations (Solanka *et al*. 2015). The resulting E–I–I models more easily produce theta oscillations with a frequency that matches that of gamma activity *in vivo* (cf. Chrobak & Buzsaki, 1998; Colgin *et al*. 2009). Although E–I models were initially motivated by the indirect connectivity between L2SCs, grid cells are found in deeper layers in which excitatory cells are likely to communicate directly with one another (Dhillon & Jones, 2000). Moreover, while many models for grid firing have focused on L2SCs, L2PCs also have grid firing fields (Sun *et al*. 2015), and the synaptic mechanisms through which they interact may differ. When E–I models are extended to include structured connectivity between excitatory neurons in addition to structured E–I interactions they continue to generate grid firing patterns (Widloski & Fiete, 2014) and nested gamma oscillations (Solanka *et al*. 2015). However, when these models were modified further so that inhibitory connectivity was random and only excitatory connectivity was structured they were unable to generate stable grid firing fields (Solanka *et al*. 2015). We suspect this results from the requirement for precise tuning of connections in continuous attractor networks based on structured excitation (cf. Seung *et al*. 2000).

The strong theta frequency modulation of activity in the MEC raises the question of how attractor states might be maintained during phases of the theta cycle in which activity is suppressed. In principle if activity is suppressed for a sufficient duration then when activity resumes the network has no memory of the location of the previous bump. The spatial representation necessary for path integration is then lost. This loss of bump stability can be prevented by synaptic or intrinsic conductances with slow dynamics (Navratilova *et al*. 2012; Pastoll *et al*. 2013; Solanka *et al*. 2015). For example, on the start of each new theta cycle the residual excitatory NMDA receptor current ensures bumps re‐form in their previous location (Fig. 4). While there is evidence that NMDA receptors in entorhinal interneurons have sufficiently slow kinetics to perform this role (Jones & Buhl, 1993), it is possible that other biophysical processes that have slow dynamics such as intracellular Ca^2+^ signalling or kinetics of the action potential after‐hyperpolarisation could also stabilise attractor states across theta cycles (Navratilova *et al*. 2012). Alternatively, theta modulation may not completely inactivate entorhinal networks, in which case bump location could be maintained through neurons that remain active across the full theta cycle. Further experimental testing of these ideas will require a better understanding of cellular mechanisms underlying modulation of entorhinal activity during theta states.

## Noise enables independent control of theta nested gamma oscillations and grid firing by modulation of excitatory–inhibitory interactions

Because E–I models account for rate coded grid computation and gamma frequency network activity, they provide an opportunity to investigate relationships between these phenomena. Many cognitive functions, in addition to spatial computation by grid networks, are associated with modulation of gamma activity (Uhlhaas & Singer, 2012). In turn, both cognitive function and gamma activity correlate with changes in E–I interactions. However, the causal relationships between the strength of excitatory and inhibitory synapses, gamma oscillations and computations that might underlie key cognitive functions have been difficult to establish. Systematic investigation of E–I models suggests that these relationships are complex (Solanka *et al*. 2015). First, nested gamma oscillations and grid firing are both promoted by an optimal level of noise within a network. If noise is too low seizure‐like states that suppress grid firing tend to emerge, whereas if noise is too high grid fields drift and gamma becomes less coherent. Second, intermediate noise levels maximise the range of excitatory and inhibitory synaptic strengths that support grid firing. Third, gamma activity is a poor predictor of grid firing. Thus, varying the strength of inhibitory or excitatory connections can tune the frequency and power of gamma oscillations without affecting grid firing. Fourth, tuning of intrinsic connections could be used to modulate oscillation‐based codes while maintaining grid firing, for example to determine the response of downstream neurons to convergent input from different grid modules. Thus, synchronisation of gamma activity between grid modules might promote, and discordant tuning of gamma activity between modules might oppose, downstream integration. Therefore, the potential for independent control of gamma oscillations and grid firing, even when both phenomena arise from a common circuit mechanism, has implications for physiological and pathological states of MEC circuits.

## Conclusion

Because multiple abstract models are able to produce grid‐like periodic spatial firing patterns, additional experimental constraints are required to establish mechanisms used by the brain to generate grid firing. We have considered evidence that continuous attractor networks that use velocity inputs to compute grid codes for location can be implemented through E–I interactions that are consistent with known properties of microcircuits in the MEC. When implemented with realistic neuronal dynamics these models also account for theta nested gamma oscillations, although so far they are unable to explain theta phase precession in two dimensions without incorporation of additional mechanisms for path integration. Critical future tests of continuous attractor network hypotheses for grid firing include evaluation of predictions for the firing patterns and connectivity of excitatory and inhibitory cell populations. E–I models make further assumptions concerning integration of velocity signals (Pastoll *et al*. 2013), error correction by place and border input (Guanella *et al*. 2007; Sreenivasan & Fiete, 2011; Pastoll *et al*. 2013; Hardcastle *et al*. 2015) and sources of tonic drive (Burak & Fiete, 2009; Bonnevie *et al*. 2013; Pastoll *et al*. 2013) that we have not considered here. Experimental evidence for how these signals are integrated by MEC circuits will further constrain possible models. Progress in establishing experimentally constrained models for spatial representation by cell populations in the MEC may serve as a proof of principle for understanding cellular and synaptic mechanisms for high‐level computations by cortical circuits in general.

## Additional information

### Competing interests

None declared.

### Funding

This work was supported by grants to M.F.N. from the BBSRC (Bb/L010496/1 and Bb/1022147/1).
